# Time to death and its predictors among neonates with seizure in North West Ethiopia

**DOI:** 10.1038/s41598-025-98628-2

**Published:** 2025-07-29

**Authors:** Tamene Fetene Terefe, Tamiru Alene, Manay Ayalneh, Nigatu Dessalegn, Yosef Aragaw Gonete, Baye Tsegaye Amlak, Fisha Alebel GebreEyesus, Alemayehu Wondie, Melaku Bimerew

**Affiliations:** 1 Department of Pediatrics and Child Health Nursing, Injibara University, Injibara, Ethiopia; 2 Department of Midwifery, Debark University, Debark, Ethiopia; 3 Department of Nursing, Debre Markos University, Debre Markos, Ethiopia; 4 Department of Nursing, Wolkite University, Wolkite, Ethiopia; 5 Department of Biomedical Science, Wolkite University, Wolkite, Ethiopia

**Keywords:** Neonatal seizure, Time to death, Ethiopia, Health care, Medical research

## Abstract

Neonatal seizures were associated with significant rates of mortality; in which about one-third of the neonates with seizure ending up with death. Despite this, the time to death and its predictors among neonates with seizure has not been investigated; especially in Ethiopia. To determine the time to death and its predictors among neonates with seizure in public hospitals of Awi zone, Northwest Ethiopia. A multicenter prospective follow-up study was conducted in public hospitals of Awi zone on 263 neonates with seizure. Descriptive statistics, Kaplan–Meier curve, Nelson–Aalen curves, and log-rank tests were employed to describe the time to death and to assess the risk of mortality among different covariates. The Cox proportional hazards model was used to identify the predictors of time to death. AHR with 95% CI was used to identify significant predictor variables, and a statistical significance was declared at *p*-value < 0.05. A total of 263 neonates with seizure were followed for a total of 1334.3 person-days, and the incidence of mortality was found to be 22.5 per 1000 person-day observations (95% CI = 14.0–29.6). The median time to death was 3 days (IQR = 2–5 days). Birth trauma (AHR = 3.9, 95% CI = 1.5–10.6), neonatal sepsis (AHR = 3.4, 95% CI = 1.1–10.8), hypoglycemia (AHR = 3.2, 95% CI = 1.1–9.3), and tonic type seizure (AHR = 4.5, 95% CI = 1.3–15.6) were statistically significant predictors of early mortality in neonates with seizure. This study revealed that the incidence of in-hospital mortality among neonates with seizure to be high and the median time to death to be short; and the predictors of early mortality were identified. Early detection and appropriate management of neonates having birth trauma, sepsis, and hypoglycemia might be helpful.

**Supplementary Information:**

The online version contains supplementary material available at 10.1038/s41598-025-98628-2.

## Introduction

A neonatal seizure can be defined as an emergency medical condition that occurs due to sudden, transient, and excessive neuronal activity in the brain of a neonate; leading to electroencephalographic abnormalities and/or overt clinical features like change in behavior, motor, or autonomic function^1–3^. Neonatal seizures could be electrographic-only, electro-clinical, or clinical-only. Electrographic-only seizures are characterized by abnormal electroencephalography (EEG) without any overt clinical signs; while electro-clinical seizures are characterized by both abnormal electroencephalography and identifiable clinical manifestations. In the absence of EEG, those overt manifestations might be used to diagnose neonatal seizures; and in this case, they would be reported as clinical-only seizures^1,4,5^. On the other hand, neonatal seizures could be classified as subtle, tonic, clonic, myoclonic, or mixed depending on their clinical presentations^2,3,6^.

Neonatal seizures are commonly caused by hypoxic-ischemic encephalopathy, infections of the central nervous system, acute metabolic disturbances, and intracranial hemorrhages^7^. Seizures are the most common neurological emergencies in neonates; with an estimated average global incidence of 1.0–5.0 per 1000 live births in full-term infants and may be even higher among preterm neonates^1,8^. Regionally, the incidence of neonatal seizure had been reported to be 0.4 cases per 1000 live births among full-term neonates and 0.2–1 cases per 1000 live births among preterm neonates in the United States^9^. Similarly, the incidence of neonatal seizure had been reported to be 0.81 cases per 1000 live births in Sweden^10^, and 2.29 cases per 1000 live births in Italy^11^. However, the burden of neonatal seizures in low and middle-income countries is much higher than the estimates from high-income countries; with an estimated incidence of 36–90 cases per 1000 live births^12^. A recent study from Ethiopia revealed that the incidence of neonatal seizure is 35 per 1000 neonate-day observations^13^, and a study from Kenya indicated that the incidence of neonatal seizure is as high as 39.5 per 1000 live births^14^.

Neonatal seizures are major causes of morbidity in infants. Full recovery from neonatal seizure had been reported only in 25–40% of the affected neonates^1^. Moreover, a significant number of survivors were affected by long-term complications like neurodevelopmental delay (30–50%), cerebral palsy (15–30%), and epilepsy (20–35%)^1,15,16^. On the worst side, neonatal seizures were associated with significant rates of mortality; in which about one-third of the neonates with seizure ended up with death^17^. A study from Ethiopia indicates that nearly 20% of neonates with seizure would die during their neonatal period and about 10.6% would have neurologic complications during their discharge from neonatal intensive care units^18^.

Literatures had been indicated that most seizure-related neonatal deaths occur early during the acute illness^17,18^. Hence, assessing the time to death and its predictors among neonates with seizure would help to identify the median time of death and predictors of early mortality. This in turn will help to develop factor-oriented strategies to reduce the early mortality among the victims of neonatal seizure. Previously, only a few studies had been conducted to assess the short-term outcomes of neonatal seizure in Ethiopia. Besides, the time to death and its predictors among neonates with seizure had not been investigated. Therefore, this study aimed to assess the time to death and its predictors among neonates with neonatal seizure in Awi zone, Ethiopia.

## Methods and materials

### Study area, design, and period

This study was conducted in Awi zone, Amhara region, Ethiopia; which is about 426 km away from Addis Ababa, the capital city of Ethiopia. Based on the 2021 census, this zone had a total population of 1,342,324; of whom 51% were females. The zone has a total of 52 health institutions; of which 47 are health centers, 4 are primary hospitals, and 1 is a general hospital. All of the hospitals (Injibara General Hospital, Agew Gimjabet Primary Hospital, Dangila Primary Hospital, Changi Primary Hospital, and Jawi Primary Hospital) provide neonatal admission services. Therefore, this multicenter prospective follow-up study was conducted in those hospitals from January 1-2023 to December 31-2023.

### Population and eligibility criteria

All neonates who were admitted to the NICUs of Awi Zone Public Hospitals with a diagnosed neonatal seizure were the source population, and all neonates who were admitted to those institutions with a diagnosed of neonatal seizure from January 1-2023 to December 31-2023 were the study population. However, neonates with complex malformations and neonates who were referred from Awi zone Public Hospitals were excluded from this study.

### Sample size and sampling technique

All neonates who have been admitted in the NICUs of Awi zone public hospitals and diagnosed as having neonatal seizure during the follow-up period and passed the eligibility criteria were samples (census method was used). During the study period (January 1-2023 to December 31-2023), a total of 5352 neonates were admitted to the NICUs of Awi Zone public hospitals. Of these, 281 neonates were diagnosed as having seizure. One neonate was diagnosed as having complex malformations, and 17 neonates were inter-hospital referrals between Awi zone public hospitals. Therefore, the final sample size of this study was 263.

### Variables of the study

*Dependent variable*: Time to death of neonates with seizure.

*Independent variables*:

- **Maternal socio-demographic factors** (age, residence, educational status, religion, marital status, and family size).
- **Baseline characteristics of neonates** (sex, age, general appearance, gestational age, birth weight, crying at birth, APGAR score, respiratory distress, status of consciousness, birth trauma, and perinatal asphyxia).
- **Obstetric characteristics of mothers** (parity, ANC follow-up, birth preparedness, place of delivery, mode of delivery, and history of bad obstetrics).
- **Seizure characteristics** (type of seizure, episodes of seizure attack, seizure onset, type of anticonvulsant therapy, and seizure control).

*Operational definitions*

#### Time to death

Is calculated from the time of seizure diagnosis till the death of the neonate.

#### Censored

Neonates who did not die during the follow-up period, including lost follow-up, referred to other health institution, discharged, or still admitted beyond 28 days of neonatal age.

#### Event

Neonates who died during the follow-up time.

#### Neonate

newborn from birth to 28 days old.

#### Neonatal seizure

Neonates were diagnosed as having seizure “clinically” if they have one or more signs of provoked or unprovoked seizure that have been witnessed by experienced physicians. These signs include: (1) focal or generalized sustained muscle stiffening or contractions, (2) regular rhythmic jerking, (3) epileptic spasms, (4) myoclonic jerks, (5) autonomic changes, (6) behavioral arrests, and (7) automatisms such as abnormal oral-buccal-lingual movements (lip smacking, chewing, tongue movements), ocular signs (fluttering, rolling, staring, and/ or deviation of the eyes), progressive movements, and complex purposeless movements^19^. After diagnosis, neonatal seizures were classified as subtle, tonic, clonic, myoclonic, or mixed according to the International League Against Epilepsy based on their clinical characteristics^20^.

#### Perinatal asphyxia

perinatal asphyxia can be defined as failure to initiate and sustain breathing at birth or having an APGAR score of < 7 at 5 minutes^21,22^. In this study, neonates having either gasping, not breathing at all, breathing rate < 30 breaths per minute at birth, and/ or APGAR score of < 7 at 5 minutes were considered as having perinatal asphyxia; and the diagnosis was made by experienced physicians.

### Data collection methods, tools, procedures, and quality control

A pre-tested and structured questionnaire and data extraction checklist were used to collect the data. Face-to-face interviews were used to collect maternal socio-demographics. Obstetrics and baseline clinical characteristics of the neonates were recorded on the day of diagnosis of neonatal seizure. Then, the neonates were followed for a maximum of 28 days; or until discharged, died, referred, or lost to follow-up. Seizure patterns, episodes of attacks, treatment & outcome-related variables were recorded at the end of follow-up period. One BSc pediatric nurse supervisor and two BSc pediatric nurse data collectors were recruited in each hospital to collect the data. Pre-testing of the data collection tool, training of the data collectors and supervisors, close monitoring and cross-checking, coding, double data entry, and cross-tabulation before analysis were employed to control the quality of the data.

### Data processing, analysis, and presentation

After coding, data were entered into EpiData version 4.6.0.6; and exported to STATA version 17 for analysis. Descriptive statistics were used to describe the socio-demographic characteristics, obstetrics, clinical profiles, and seizure characteristics of the study participants. The hazard of death among neonates admitted with seizure across different categories of the covariates was compared by using the Nelson Aalen curves and log-rank tests. Kaplan–Meier survival estimate curve was used to plot the survival probability. The Cox proportional hazards model was used to identify the predictors of time to death. First, bi-variable cox-regression was employed and variables with a *p*-value of less than 0.25 were further entered into multivariable cox regression model. Hazard ratio with its 95% confidence interval and *p*-values was used to measure the strength of the association and identify statistically significant results. Variables having *p*-value < 0.05 were considered as having statistically significant association. The Cox- proportional hazards model assumption was checked graphically by the log minus log function; and statistically by Schoenfeld residual (global) test. Adjusted hazard ratio (AHR) with 95% confidence interval was used to identify significant predictive variables and a statistical significance was declared at *p*-value < 0.05. Finally, results have been presented with texts, tables, and curve graphs.

## Results

### Socio-demographic characteristics of mothers of neonates with seizure

The mean age ± standard deviation (SD) of mothers of the study participants was 27.41 ± 5.85 years. Nearly half 125(47.5%) of the mothers were aged less than or equal to 25 years. More than half (54.4%) of the mothers were rural residents. Majority (79.8%) of the mothers were followers of Orthodox Christianity. About 95.1% and 72.6% of the mothers were married and live in a household with less than 5 family members respectively (Table 1).

**Table 1:** Socio demographic characteristics of mothers of neonates with seizure at NICU of Awi zone public hospitals, Northwest Ethiopia, 2023(*N* = 263).

Variables		Frequency (*n*)	Percentage (%)
Age	≤ 25	125	47.5
	25–35	107	40.7
	≥ 35	31	11.8
Residence	Urban	120	45.6
	Rural	143	54.4
Religion	Orthodox	210	79.8
	Muslim	24	9.1
	Protestant	15	5.7
	Others*	14	5.3
Marital status	Married	250	95.1
	Divorced	8	3.0
	Widowed	3	1.1
	Single	2	0.8
Occupation	House wife	99	37.6
	Farmer	102	38.8
	Government employee	31	11.8
	Merchant	17	6.5
	Others*	14	5.32
Family size	< 5	191	72.6
	≥ 5	72	27.4

*Private employee, daily laborer

### Baseline clinical characteristics of the neonates

Among 263 neonates enrolled, 50.6% were females; and 52.9% were aged less than 1 day at admission. Nearly two-thirds (63.1%) of neonates had normal birth weight; with a mean ± SD birth weight of 2797 ± 646.9 g. About 66.5% of the neonates were cried immediately after delivery; while one-fourth (25.1%) of the neonates were asphyxiated (Table 2 and supplementary Table 1).

**Table 2:** Base line clinical characteristics of the neonates diagnosed with seizure at NICU of Awi zone public hospitals, North West Ethiopia, 2023 (*N* = 263).

Variable		Frequency (*n*)	Percentage (%)
Sex	Male	130	49.4
	Female	133	50.6
Birth weight	Low birth weight (< 2500gms)	87	33.1
	Normal birth weight ([2500–4000)grams)	166	63.1
	Macrosomia (≥ 4000gms)	10	3.8
Cry at delivery	Immediately	175	66.5
	Delayed	88	33.5
5th minute APGAR score	≤ 7	115	43.7
	8–10	135	51.3
	Unknown*	13	4.9
Fetal distress	Yes	69	26.2
	No	194	73.8
Mental status	Alert	134	51.0
	Lethargic	103	39.2
	Comatose	26	9.9
Birth trauma	Yes	43	16.4
	No	220	83.7
Perinatal asphyxia	Yes	66	25.1
	No	197	74.9
Sepsis	Yes	41	15.6
	No	222	84.4
Blood glucose	< 40 mg/dl	88	33.5
	40-125 mg/dl	158	60.1
	> 125 mg/dl	17	6.5
Hypoxic ischemic encephalopathy	Yes	104	39.5
	No	159	60.5
Meningitis	Yes	24	9.1
	No	239	90.9
Acute bilirubin encephalopathy	Yes	6	2.3
	No	257	97.7
Sucking reflex	Complete	97	36.9
	Not sustain	155	58.9
	Absent	11	4.2
Moro reflex	Complete	144	54.8
	Incomplete	109	41.4
	Absent	10	3.8
Palmar grasp reflex	Strong	125	47.5
	Weak	129	49.1
	Absent	9	3.4

APGAR, appearance, pulse, grimace, activity, respiration

*Might be due to home delivery

### Obstetrics characteristics of mothers

Most of the mothers (92%) had received antenatal care; of these, about 47.1% had received ANC follow-up at least four times. About 24% of mothers had bad obstetrics history; of them, 14.8% of the mothers had newborn death, 10.6% had a history of abortion, and 9.9% had a history of stillbirth. About 17.5% of the mothers had hypertensive disorders of pregnancy during the current pregnancy. Most (95.1%) of the mothers had given birth at health institutions, and more than half (59.3%) had given birth spontaneously (Table 3).

**Table 3:** Obstetric characteristics of mothers of neonates with seizure at NICU of Awi zone public hospitals, North West Ethiopia, 2023(*N* = 263).

Variables		Frequency	Percentage
Parity	Primipara	119	45.2
	Multipara	144	54.8
ANC follow up	Yes	242	92.0
	No	21	8.0
Number of ANC care visits	> 4 times	118	44.9
	≥ 4 times	124	47.1
Gestational age in weeks	≥ 37weeks	79	30.0
	37–42 weeks	178	67.7
	≥ 42 weeks	6	2.3
Duration of labor	Precipitated	42	16.0
	Normal	206	78.3
	Prolonged	15	5.7
Place of delivery	Health institution	250	95.1
	Home	13	4.9
Mode of delivery	SVD	156	59.3
	Assisted SVD(vacuum or forceps)	75	28.5
	Caesarean section	32	12.2
History of bad obstetrics	Yes	63	24.0
	No	200	76.0
History of newborn death	Yes	39	14.8
	No	224	85.2
History of abortion	Yes	28	10.6
	No	235	89.4
History of still birth	Yes	26	9.9
	No	237	90.1
Hypertension during current pregnancy	Yes	46	17.5
	No	217	82.5
Type of hypertension during current pregnancy	Preeclampsia	39	14.8
	Eclampsia	7	2.7

### Patterns, treatments & outcomes of neonatal seizure

Out of 5352 NICU admissions over one year period, there were about 263 (4.9%) neonatal seizure cases. The most frequently observed seizure (58.6%) was a subtle seizure; followed by a tonic seizure (21.3%). About 70.7% of the neonates experienced their first seizure within 72 h of birth. Nearly three-fourths (70.3%) of neonates had multiple episodes of seizures. All neonates had received anticonvulsant therapy. Phenobarbitone was prescribed for about (15.2%) of neonates, and 62.0% of the neonates received more than one anticonvulsant drug. Of these, 36.5% used two drug combinations (calcium gluconate and phenobarbital); and 25.5% used three drug combinations (calcium gluconate, phenobarbital, and phenytoin). Well-controlled seizures were seen in 72.6% of the neonates. About two third (66.9%) of the neonates stay in the ward for less than or equal to 7 days. Regarding treatment outcomes, 68.1% of the neonates were discharged with improvement and 11.4% were died (Table 4).

**Table 4:** Patterns, time onset, episodes of attacks, treatment & outcome related of neonates with seizure at NICU of Awi zone public hospitals, North West Ethiopia, 2023(*N* = 263).

Variables		Frequency	Percentage
Type of seizure	Sublte	154	58.56
	Tonic	56	21.29
	Clonic	19	7.22
	Myoclonic	15	5.7
	Mixed	19	7.22
Episodes of seizure	single episodes	86	32.7
	Multiple episodes	177	67.3
Seizure onset	≤ 72 h	186	70.7
	> 72 h	77	29.3
Anticonvulsant therapy	Yes	263	100.0
Type of anticonvulsant therapy started	Phenobarbital	40	15.2
	Calcium gluconate	22	8.4
	Phenytoin	25	9.5
	Diazepam	13	4.9
	Mixed	163	62.0
Drugs used during mixed therapy	Two (Phenobarbital AND calcium gluconate)	96	36.5
	Three (Phenobarbital AND calcium gluconate AND Phenytoin)	67	25.5
Seizure control	Well controlled	198	75.29
	Poorly controlled	65	24.71
Hospital length of stay	≤ 7 days	173	65.8
	7–14 days	77	29.2
	14–21 days	7	2.7
	≥ 21 days	6	2.3
Outcomes of treatment	Died	30	11.4
	Referred	29	11.0
	Improved	179	68.1
	left against medical advice	15	5.7
	Still on treatment	10	3.8

### Time to death of neonates with seizure

A total of 263 neonates admitted with seizure were followed for a median follow-up period of 4 days (IQR: 2–7 days), with a minimum of half a day and a maximum of 23 days follow-up period. Of the total study subjects, 30(11.41% (95% CI = 8.0 – 15.0%)) neonates died during the study period. The incidence rate of death was 22.5 (95% CI = 14.0–29.6) per 1000 person-days of observation, with a total follow-up time of 1334.3 person-days of observation. Regarding the time of death, 7(23.3%), 13(49.4%), 16(60.8%), and 22 (83.7%) of deaths occurred within the 2nd, 3rd, 4th and 7th days of follow up respectively; and the median time to death was found to be 3 days with an IRQ of 2–7days.

**Fig. 1:**
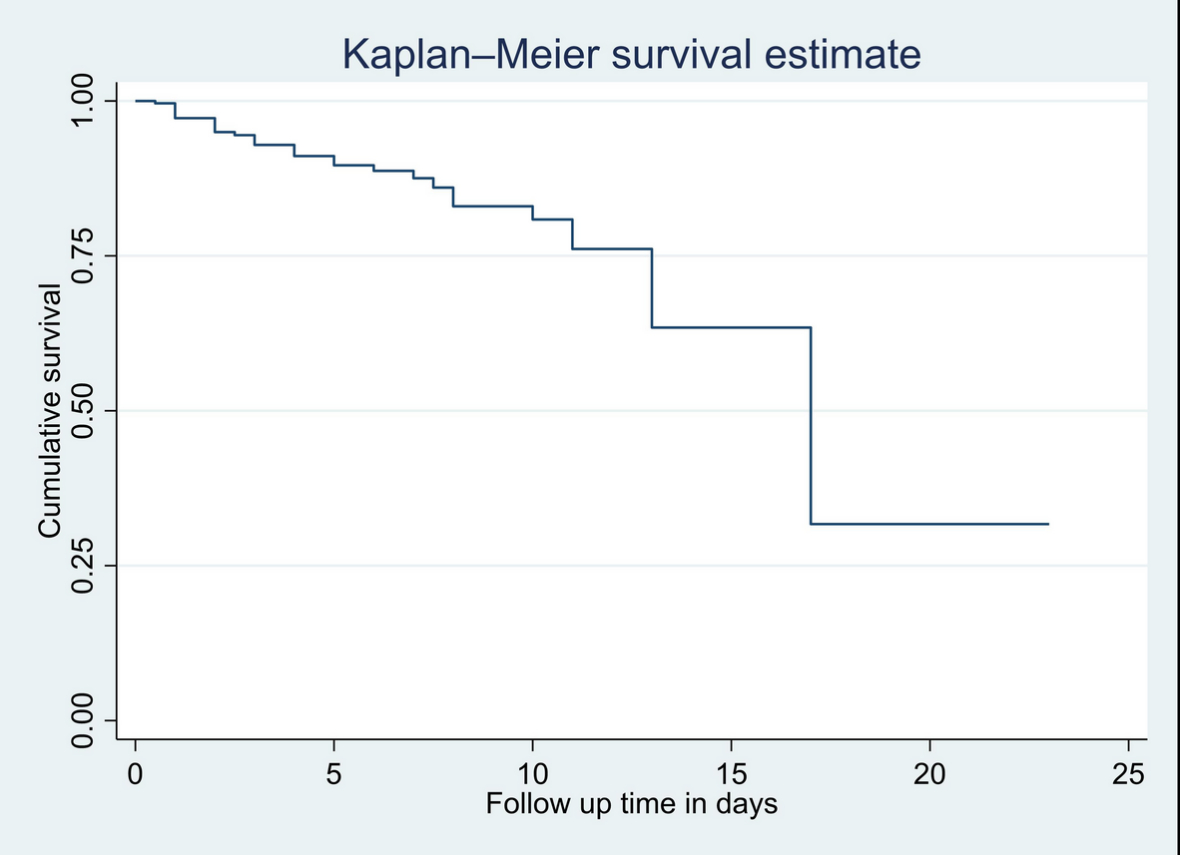
Kaplan–Meier cumulative survival estimate of neonates with seizure at NICUs of public hospitals in Awi zone, northwest Ethiopia, 2023 (*N* = 263).

Regarding survival Probability, the cumulative survival among neonates with seizure on the 1st, 3rd, 6th, 12th, and 23rd day of admission were 0.996 (95% CI 0.972, 0.999), 0.94% (95% CI 0.9, 0.97), 0.89% (95% CI 0.83, 0.93), 0.78 (95%CI 0.68,0.85), and 0.52% (95% CI 0.2, 0.57), respectively (Fig. 1).

**Fig. 2:**
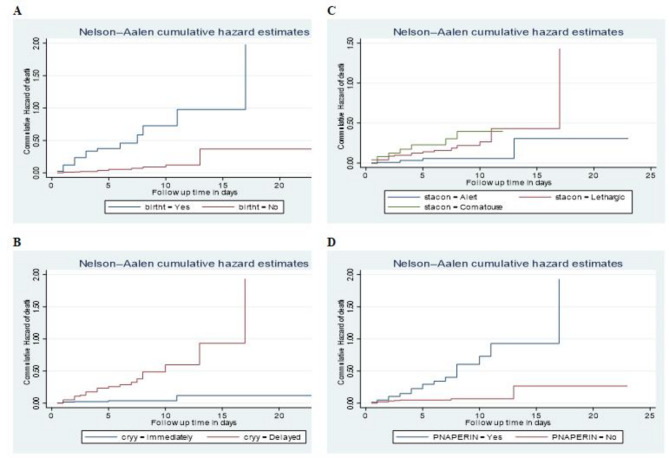
Nelson–Aalen cumulative hazard curve showing the hazards of death across different categories of the covariates (birth trauma (**A**), crying status at birth (**B**), status of consciousness at admission (**C**), and perinatal asphyxia (**D**).

The Nelson–Aalen curve shows that the hazard of death among neonates admitted with seizure differs across different baseline clinical characteristics of the neonates. Neonates with birth trauma (*p*-value < 0.001), neonates who were comatose at admission (*p*-value = 0.008), neonates whose first cry was delayed (*p*-value < 0.001), and neonates with perinatal asphyxia (*p*-value < 0.001) had a statistically significant higher hazard of death as compared to their counterparts (Fig. 2).

### Test of model fitness

**Fig. 3:**
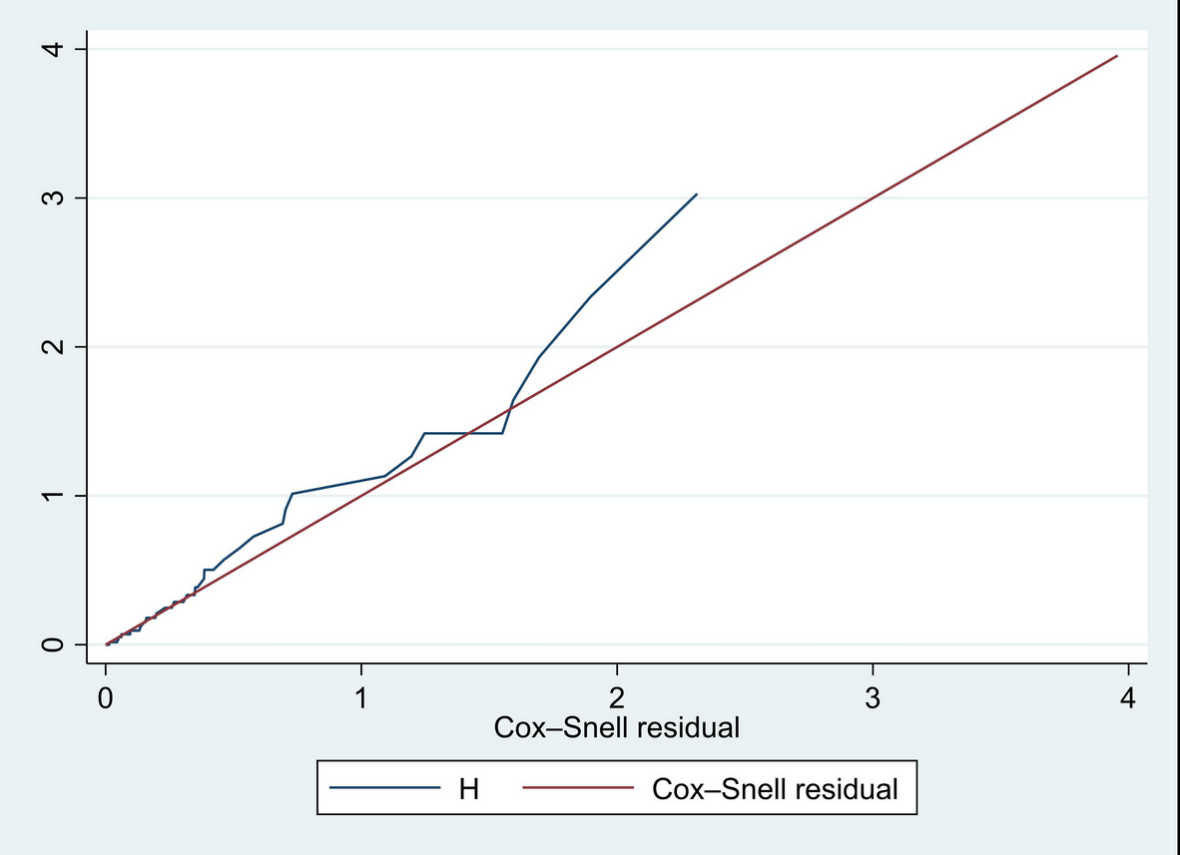
Cox-Snell residual test for overall adequacy of the model fitted for time to death among neonates with of neonatal seizure in NICUs of Public hospitals in Awi zone, Northwest Ethiopia; 2023.

The Cox proportional hazard (PH) assumption was fulfilled, as shown both statistically and graphically. Statistically, it was tested by the Schoenfeld residual test (global test, X^2^ = 12.82, *p*-value = 0.8478). Graphically, the overall goodness of fit of the Cox regression model was tested by applying the Cox-Snell residual plots. As shown in Fig. 3, the line connected with the Cox Snell residual of the Cox regression model was nearer to 45° straight lines of the origin, which showed that the model is well fitted (Fig. 3).

### Predictors of time to death among neonates with seizure

To identify predictors of time to death among neonates with seizure, initially, all the study variables^28^ were entered to the bivariable Cox proportional model. In this model, 14 variables were found to be associated with time to death at a *p*-value < 0.25; and they were considered to be candidates for the multivariable Cox proportional model.

Of the 14 variables entered to the multivariable Cox regression, 4 variables (neonates with birth trauma, having sepsis at admission, neonates with admission blood glucose value < 40 mg/dl, and neonates with tonic seizure) were found to be significant predictors of time to death among neonates with seizure.

The hazard of neonatal death was four times (AHR = 3.94, 95% CI 1.46, 10.64) higher among neonates with birth trauma as compared to their counterparts. Neonates who had Sepsis at admission had nearly three times (AHR = 3.38, 95% CI 1.06, 10.83) higher risk of death than those who did not had sepsis. The hazard of death among neonates with admission blood glucose value of < 40 mg/dl was nearly three times (AHR = 3.24, 95% CI 1.13, 9.29) as compared to neonates whose admission blood glucose value was 40–125 mg/dl. The hazard of neonatal death was nearly 4.5 times (AHR = 4.49, 95% CI 1.29, 15.61) higher for neonates who have tonic seizure as compared to neonates who have subtle seizure (Table 5).

**Table 5:** Predictors of death among neonates diagnosed with seizure at NICU of Awi zone public hospitals, North West Ethiopia, 2023(*N* = 263).

Variables		Outcome		CHR ( 95% CI)	AHR (95% CI)
		Death	Censored		
Residence	Urban	5	115	0.25(0.09,0.65)	0.56(0.18, 1.79)
	Rural	25	118	1	
Sex	Male	9	121	1	
	Female	21	112		1.55(0.56, 4.24)
Crying at birth	Immediately	6	169	1	
	Delayed	24	64	8.13(3.33 19.95)	1.24(0.35, 4.4)
Fetal distress	Yes	22	47	8.54(3.78, 19.3)	2.55(0.78, 8.36)
	No	8	186	1	
Birth trauma	Yes	18	25	7.86(3.75,16.47)	**3.94(1.46**,** 10.64)**
	No	12	208	1	
Perinatal asphyxia	Yes	20	46	1	
	No	10	187	6.45(3, 13.83)	1.3(0.34, 4.98)
Sepsis at admission	Yes	20	21	10.47(4.86, 22.56)	**3.38(1.06**,** 10.83)**
	No	10	212	1	
Blood glucose level	< 40 mg/dl	18	70	4.48(1.91,10.5)	**3.24(1.13**,** 9.29)**
	40-125 mg/dl	9	149	1	
	≥ 125 mg/dl	3	14	2.64(0.7,10)	1.75(0.36, 8.62)
Parity	Primiparity	17	102	1	
	Multipara	13	131	0.54(0.26 ,1.14)	0.59(0.36, 1.79)
Episodes of seizure	Single episode	2	84	1	
	Multiple episodes	28	149	7.51(1.78, 31.65)	2.64(0.5,14.08)
Type of seizure	Subtle	5	149	1	
	Tonic	19	37	8.63(3.41, 21.83)	**4.49(1.29**,** 15.61)**
	Clonic	1	18	1.49(0.18, 12.47)	1.55(0.16, 15.31)
	Myoclonic	4	11	7.1(1.98, 25.51)	0.61(0.07, 5.53)
	Mixed	1	18	1.53(0.18, 12.84)	2.8(0 0.34, 30.42)
History of ANC follow up	Yes	26	216		
	No	4	17	2.3(0.79, 6.76)	1.33(0.27, 6.53)
History of bad obstetrics	Yes	20	53		
	No	10	180	1.85(0.86, 3.99)	1.28(0.65, 3.72)
Birth weight	Low birth weight	18	69	3.81(1.75, 8.28)	2.76(0.96, 7.93)
	Normal birth weight	11	155	1	
	Macrosomia	1	9	1.13(0.14, 8.94)	1.35(0.12, 14.73)

Significant values are in bold.

## Discussion

This study aimed to assess the time to death and its predictors among neonates with seizure in public hospitals of Awi zone, Ethiopia. Accordingly, the median time to death among neonates with seizure in the study area was found to be 3 days with an IQR of 2–7 days. In this study, among 263 neonates with seizure, 30(11.41%, 95% CI 8–15) neonates were died during the follow-up period. This finding was lower than previous studies done in northern Ethiopia (21.3%)^23^, Northwest Ethiopia (19.7%)^18^, Bosnia (23%)^24^ and India (17.2%)^25^. The revealed lower mortality in this study as compared to previous Ethiopian studies might be due to differences in the study settings; as the previous studies were conducted in referral hospitals; whereas, this study was conducted in 4 primary and 1 general hospitals. In fact, neonates with serious cases would be referred to nearby referral hospitals, and the mortality rate of neonates with seizure in referral hospitals might be higher than neonates in primary and general hospitals; as exhibited in this study. Similarly, the lower mortality in this study as compared to the results of the studies in Bosnia and India might be due to the fact that these studies were done in level III NICU, where neonates with more serious cases are managed and the mortality rate for such neonates may be higher. In addition, the study done in Bosnia followed the neonates for period of one year, which may have tendency increase the mortality rate.

In this study, the incidence rate of death was found to be 22.5 per 1000 person-days of observation (95% CI = 14.0–29.6), with a total follow-up time of 1334.3 person-days. This study also revealed that about 83.7% of deaths among neonates with seizure occurred during the first week of their life. As per the author’s knowledge, there was no prior published evidence to discuss this incidence rate and time of death.

In this study, the findings of multivariable Cox regression analysis showed neonates with birth trauma, having sepsis at admission, neonates with admission blood glucose value < 40 mg/dl, and neonates with tonic seizure and were significant predictors of early death among neonates with seizure. The hazard of early mortality was nearly 4 times higher in neonates with birth trauma than in neonates without birth trauma. This finding was supported by a study conducted in the United States^26^. Birth trauma is one of the perinatal care quality indicators and^27^; these injuries can cause widespread brain damage including bleeding between the brain and the skull; which in turn leads to anemia, decreased blood flow to the brain, and/ or low oxygenation of the blood. Therefore, neonates with coexisting seizure and birth trauma would have an increased risk of early death than neonates with seizure; as revealed in this study.

In this study, having sepsis at admission was found to be a significant predictor of early death among neonates with seizure; as the hazard of early death was three times higher among neonates with sepsis than neonates without sepsis. This finding was in line with previous studies conducted in the United States^26^, Israel^28^, Iran^29^ and India^25^. In fact, infection during the neonatal period is a common cause of neonatal seizures; and neonates with seizure and infection would have a higher risk of early death^30,31^. Similarly, as per the results of this study, neonates with admission blood glucose value of < 40 mg/dl had three times higher risk of death than those whose admission blood glucose value was 40–125 mg/dl. This result was congruent with previous studies conducted in Canada^32^ India^33^ and Iran^34,35^. Metabolic disturbances such as hypoglycemia are responsible for neonatal seizures; and when such metabolic disturbances are the primary cause of neonatal seizures, they are associated with significant short-term consequences^36^. This study also revealed that neonates with tonic-type seizure were nearly five times more likely to die early than neonates with subtle-type seizure. This finding was similar to a study done at the University of Gondar Hospital^18^, Ethiopia. Evidences had showed that tonic seizure is frequently associated with intra-ventricular hemorrhage and increase the risk of death.

### Strengths and limitations of the study

This study was a multicenter prospective follow-up study; and had used robust research methods. However, it might not be free from some minor limitations. Firstly, as there were no definitive diagnostic infrastructures (EEG) for diagnosing neonatal seizure in Ethiopia, this study had used clinical-only seizures. Therefore, the reported incidence of neonatal seizure might not indicate the true incidence; and the exhibited time to death might not represent electrographic-only seizures. Secondly, due to resource constraints, the follow-up period was relatively short. Hence, this study did not reported the fate of neonates with seizure who were still on treatment for seizure. Additionally, it has not revealed the neurodevelopmental outcomes of the survivors of neonatal seizure.

## Conclusion and recommendations

This study had revealed that the incidence of in-hospital mortality among neonates with seizure is high and the median time to death is short. Despite the advancements in neonatal intensive care units, more than one in ten neonates with seizures are dying in the study area. The median time to death was 3 days, and more than eight in ten neonates with seizure are dying in the first week of their life. Birth trauma, sepsis, hypoglycemia, and tonic type seizure were significant predictors of early mortality among neonates with seizure. Therefore, a special emphasis and close follow should be given to neonates with seizure in the first 7 days of their life. Early detection and appropriate management of neonates having birth trauma, sepsis, and hypoglycemia might also be helpful to reduce seizure-related neonatal mortalities. It is also better if future researches are conducted on this issue by addressing the limitations of this study.

## Electronic supplementary material

Below is the link to the electronic supplementary material.

Supplementary Material 1

## Data Availability

The datasets supporting the conclusions of this article are provided within the manuscript or supplementary information files.
