# Supplementary material for: Time to death and its predictors among neonates with seizure in North West Ethiopia
Source: Sci Rep. 2025 Jul 29;15:27692. doi: 10.1038/s41598-025-98628-2 (PMC12307760; doi:10.1038/s41598-025-98628-2)
Supplement: Supplementary file 1 — Supplementary Material 1 [file 41598_2025_98628_MOESM1_ESM.docx]

Supplementary table 1: base line vital signs and 1^st^ minute APGAR score of neonates diagnosed with seizure at NICU of Awi zone public hospitals, North West Ethiopia, 2023(N=263)

| **Variable** | | **Frequency** | **Percentage (%)** |
| --- | --- | --- | --- |
| **Pulse rate (beats/minute)** | <100 | 34 | 12.9 |
|  | 100-160 | 180 | 68.4 |
|  | >160 | 49 | 18.6 |
| **Respiratory rate (respirations/ minute)** | <30 | 25 | 9.5 |
|  | 30-60 | 188 | 71.5 |
|  | ≥60 | 50 | 19.0 |
| **Axillary temperature (℃)** | <36.5 | 165 | 62.7 |
|  | 36.5-37.5 | 58 | 22.1 |
|  | ≥37.5 | 40 | 15.2 |
| **1^st^ minute APGAR score** | < 7 | 168 | 63.9 |
|  | 7-10 | 82 | 31.2 |
|  | Unknown* | 13 | 4.9 |
| *APGAR= Appearance, Pulse, Grimace, Activity, Respiration*  **Might be due to home delivery* | | | |
